# Facebook Use Predicts Declines in Subjective Well-Being in Young Adults

**DOI:** 10.1371/journal.pone.0069841

**Published:** 2013-08-14

**Authors:** Ethan Kross, Philippe Verduyn, Emre Demiralp, Jiyoung Park, David Seungjae Lee, Natalie Lin, Holly Shablack, John Jonides, Oscar Ybarra

**Affiliations:** 1 Psychology Department, University of Michigan, Ann Arbor, Michigan, United States of America; 2 Psychology Department, University of Leuven, Leuven, Belgium; Institut Pluridisciplinaire Hubert Curien, France

## Abstract

Over 500 million people interact daily with Facebook. Yet, whether Facebook use influences subjective well-being over time is unknown. We addressed this issue using experience-sampling, the most reliable method for measuring in-vivo behavior and psychological experience. We text-messaged people five times per day for two-weeks to examine how Facebook use influences the two components of subjective well-being: how people feel moment-to-moment and how satisfied they are with their lives. Our results indicate that Facebook use predicts negative shifts on both of these variables over time. The more people used Facebook at one time point, the worse they felt the next time we text-messaged them; the more they used Facebook over two-weeks, the more their life satisfaction levels declined over time. Interacting with other people “directly” did not predict these negative outcomes. They were also not moderated by the size of people's Facebook networks, their perceived supportiveness, motivation for using Facebook, gender, loneliness, self-esteem, or depression. On the surface, Facebook provides an invaluable resource for fulfilling the basic human need for social connection. Rather than enhancing well-being, however, these findings suggest that Facebook may undermine it.

## Introduction

Online social networks are rapidly changing the way human beings interact. Over a billion people belong to Facebook, the world's largest online social network, and over half of them log in daily [1]. Yet, no research has examined how interacting with Facebook influences subjective well-being over time. Indeed, a recent article that examined every peer-reviewed publication and conference proceeding on Facebook between 1/2005 and 1/2012 (412 in total) did not reveal a single study that examined how using this technology influences subjective well-being over time (Text S1) [2].

Subjective well-being is one of the most highly studied variables in the behavioral sciences. Although significant in its own right, it also predicts a range of consequential benefits including enhanced health and longevity [3]–[5]. Given the frequency of Facebook usage, identifying how interacting with this technology influences subjective well-being represents a basic research challenge that has important practical implications.

This issue is particularly vexing because prior research provides mixed clues about how Facebook use should influence subjective well-being. Whereas some cross-sectional research reveals positive associations between online social network use (in particular Facebook) and well-being [6], other work reveals the opposite [7], [8]. Still other work suggests that the relationship between Facebook use and well-being may be more nuanced and potentially influenced by multiple factors including number of Facebook friends, perceived supportiveness of one's online network, depressive symptomatology, loneliness, and self-esteem [9], [10], [11].

So, how does Facebook usage influence subjective well-being over time? The cross-sectional approach used in previous studies makes it impossible to know. We addressed this issue by using experience-sampling, the most reliable method for measuring in-vivo behavior and psychological experience over time [12]. We text-messaged participants five times per day for 14-days. Each text-message contained a link to an online survey, which participants completed using their smartphones. We performed lagged analyses on participants' responses, as well as their answers to the Satisfaction With Life Questionnaire (SWLS) [13], which they completed before and immediately following the 14-day experience-sampling period, to examine how interacting with Facebook influences the two components of subjective well-being: how people feel (“affective” well-being) and how satisfied they are with their lives (“cognitive” well-being) [14], [15]. This approach allowed us to take advantage of the relative timing of participants' natural Facebook behavior and psychological states to draw inferences about their likely causal sequence [16]–[19].

## Methods

### Participants

Eighty-two people (*M*_age_ = 19.52, *SD*_age_ = 2.17; 53 females; 60.5% European American, 28.4% Asian, 6.2% African American, and 4.9% other) were recruited for a study on Facebook through flyers posted around Ann Arbor, Michigan. Participants needed a Facebook account and a touch-screen smartphone to qualify for the study. They received $20 and were entered into a raffle to receive an iPad2 for participating.

### Ethics Statement

The University of Michigan Institutional Review Board approved this study. Informed written consent was obtained from all participants prior to participation.

### Materials and Procedure

#### Phase 1

Participants completed a set of questionnaires, which included the SWLS (*M* = 4.96, *SD* = 1.17), Beck Depression Inventory [20] (*M* = 9.02, *SD* = 7.20), the Rosenberg Self-Esteem Scale [21] (*M* = 30.40, *SD* = 4.96), and the Social Provision Scale [22] (*M* = 3.55, *SD* = .34), which we modified to assess perceptions of Facebook support. We also assessed participants' motivation for using Facebook by asking them to indicate whether they use Facebook “to keep in touch with friends (98% answered yes),” “to find new friends (23% answered yes),” “to share good things with friends (78% answered yes),” “to share bad things with friends (36% answered yes),” “to obtain new information (62% answered yes),” or “other: please explain (17% answered yes).” Examples of other reasons included chatting with others, keeping in touch with family, and facilitating schoolwork and business.

#### Phase 2

Participants were text-messaged 5 times per day between 10am and midnight over 14-days. Text-messages occurred at random times within 168-minute windows per day. Each text-message contained a link to an online survey, which asked participants to answer five questions using a slider scale: (1) How do you feel right now? (*very positive* [0] to *very negative* [100]; *M* = 37.47, *SD* = 25.88); (2) How worried are you right now? (*not at all* [0] to *a lot* [100]; *M* = 44.04, *SD* = 30.42); (3) How lonely do you feel right now? (*not at all* [0] to *a lot* [100]; *M* = 27.61, *SD* = 26.13); (4) How much have you used Facebook since the last time we asked? (*not at all* [0] to *a lot* [100]; *M* = 33.90, *SD* = 30.48); (5) How much have you interacted with other people “directly” since the last time we asked? (*not at all* [0]to *a lot* [100]; *M* = 64.26, *SD* = 31.11). When the protocol for answering these questions was explained, interacting with other people “directly” was defined as face-to-face or phone interactions. An experimenter carefully walked participants through this protocol to ensure that they understood how to answer each question and fulfill the study requirements.

Participants always answered the affect question first. Next the worry and loneliness questions were presented in random order. The Facebook use and direct social interaction questions were always administered last, again in random order. Our analyses focused primarily on affect (rather than worry and loneliness) because this affect question is the way “affective well-being” is typically operationalized.

#### Phase 3

Participants returned to the laboratory following Phase 2 to complete another set of questionnaires, which included the SWLS (*M* = 5.13, *SD* = 1.26) and the Revised UCLA Loneliness Scale [23] (*M* = 1.69, *SD* = .46). Participants' number of Facebook friends (*M* = 664.25, *SD* = 383.64) was also recorded during this session from participants' Facebook accounts (Text S2).

## Results

### Attrition and compliance

Three participants did not complete the study. As the methods section notes, participants received a text message directing them to complete a block of five questions once every 168 minutes on average (the text message was delivered randomly within this 168-minute window). A response to any question within a block was considered “compliant” if it was answered *before* participants received a subsequent text-message directing them to complete the next block of questions. Participants responded to an average of 83.6% of text-messages (range: 18.6%–100%). Following prior research [24], we pruned the data by excluding all of the data from two participants who responded to <33% of the texts, resulting in 4,589 total observations. The results did not change substantively when additional cutoff rates were used.

### Analyses overview

We examined the relationship between Facebook use and affect using multilevel analyses to account for the nested data structure. Specifically, we examined whether T_2_ affect (i.e., How do you feel *right now?*) was predicted by T_1–2_ Facebook use (i.e., How much have you used Facebook *since the last time we asked?*), controlling for T_1_ affect at level-1 of the model (between-day lags were excluded). Note that although this analysis assesses Facebook use at T_2_, the question refers to usage between T_1_ and T_2_ (hence the notationT_1–2_). This analysis allowed us to explore whether Facebook use during the time period separating T_1_ and T_2_ predicted changes in affect over this time span.

When non-compliant cases were observed, we used participants' responses to the last text message they answered to examine the lagged effect of Facebook use on well-being to maximize power. So, if we were interested in examining whether T_2–3_ Facebook use predicted T_3_ Affect controlling for T_2_ Affect, but did not have data on T_2_ Affect, then we used T_1_ Affect instead. Excluding trials in which participants did not respond to the previous texts (rather than following the aforementioned analytical scheme) did not substantively alter any of the results we report.

Significance testing of fixed effects was performed using chi-squared distributed (df = 1) Wald-tests. All level-1 predictors were group-mean centered, and intercepts and slopes were allowed to vary randomly across participants (see Table 1 for zero-order correlations). We tested for moderation by examining whether each moderator variable was related to the slope of T_1–2_ Facebook use when predicting T_2_ affect, controlling for T_1_ affect.

**Table 1:** Within-person and between-person zero-order correlations.

	Experience-sampled variables					Pre/post experience sampling	
	Affect	Worry	Loneliness	Facebook use	Direct contact	Pre life satisfaction	Post life satisfaction
Affect	–	.53***	.50***	.14***	−.29***	–	–
Worry	.77***	–	.37***	.17***	−.23***	–	–
Loneliness	.68***	.66***	–	.22***	−.40***	–	–
Facebook Use	.07	.13	.22*	–	−.24***	–	–
Direct Contact	−.28*	−.09	−.39***	.26*	–	–	–
Pre Life Satisfaction	−.55***	−.41***	−.40***	−.05	.29**	–	–
Post Life Satisfaction	−.66***	−.51***	−.48***	−.18	.23*	.86***	–

*Note*. Correlations above the dashed diagonal line represent within-person correlations obtained from multi-level analyses. Correlations below the dashed diagonal line represent between-person correlations.

* *p*<.05.

** *p*<.01.

*** *p*<.001.

Data from one person who scored 4SDs above the sample mean on the BDI were excluded from the BDI moderation analyses; data from one person who scored 4SDs above the sample mean on number of Facebook friends were excluded from the moderation analyses based on Facebook friends.

The relationship between mean Facebook use and life satisfaction was assessed using OLS regressions because these data were not nested. Both unstandardized (*B*) and standardized (*β*) OLS regression coefficients are reported (see Text S3).

### Facebook use and well-being

#### Affective well-being

**Figure 1:**
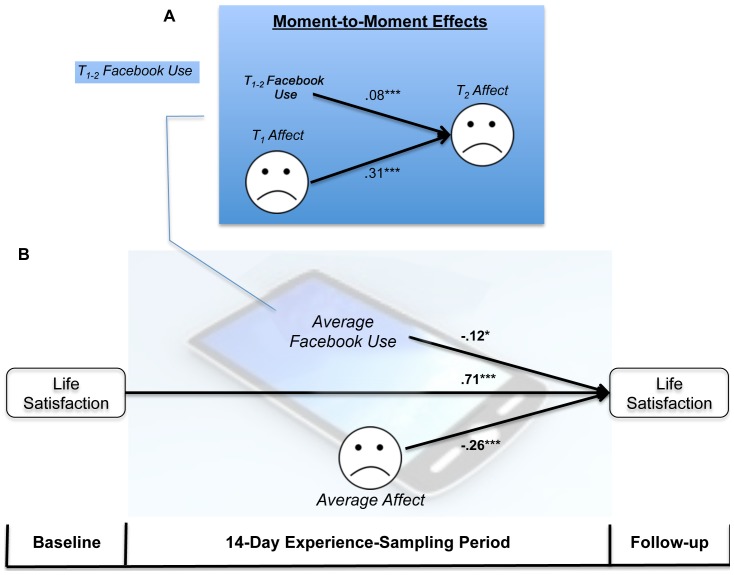
Facebook use predicts declines in affect and life satisfaction over time. Interacting with Facebook during one time period (Time_1–2_) leads people to feel worse later on during the same day (T_2_) controlling for how they felt initially (T_1_); values are regression weights from multilevel analyses (Panel A). Average Facebook use over the course of the 14-day experience-sampling period predicts decreases in life satisfaction over time; values are standardized regression weights from OLS regression analysis (Panel B). *p<.05, ** *p*<.01, ***p<.001.

We examined whether people's tendency to interact with Facebook during the time period separating two text messages influenced how they felt at T_2_, controlling for how they felt at T_1_. Nested time-lag analyses indicated that the more people used Facebook the worse they subsequently felt, *B* = .08, *χ^2^* = 28.90, *p*<.0001, (see Figure 1, top). The reverse pathway (T_1_ Affect predicting T_1–2_ Facebook use, controlling for T_0–1_ Facebook use) was not significant, *B* = −.005, *χ^2^* = .05, *p* = .82, indicating that people do not use Facebook more or less depending on how they feel (see Text S4, S5).

#### Cognitive well-being

To examine how Facebook use influenced “cognitive well-being,” we analyzed whether people's average Facebook use over the 14-day period predicted their life satisfaction at the end of the study, controlling for baseline life satisfaction and average emotion levels over the 14-day period. The more participants used Facebook, the more their life satisfaction levels declined over time, *B* = −.012, *β* = −.124, *t*(73) = −2.39, *p* = .02, (see Figure 1, bottom).

#### Alternative explanations

An alternative explanation for these results is that any form of social interaction undermines well-being. Because we also asked people to indicate how frequently they interacted with other people “directly” since the last time we text messaged them, we were able to test this idea. Specifically, we repeated each of the aforementioned analyses substituting “direct” social interaction for Facebook use. In contrast to Facebook use, “direct” social interaction did not predict changes in cognitive well-being, *B* = −.006, *β* = −.059, *t*(73) = 1.04, *p* = .30, and predicted *increases* (not decreases) in affective well-being, *B* = −.15, *χ^2^* = 65.30, *p*<.0001. Controlling for direct social interaction did not substantively alter the significant relationship between Facebook use and affective well-being, *B* = .05, *χ^2^* = 10.78, *p*<.01.

Another alternative explanation for these results is that people use Facebook when they feel bad (i.e., when they are bored lonely, worried or otherwise distressed), and feeling bad leads to declines in well-being rather than Facebook use per se. The analyses we reported earlier partially address this issue by demonstrating that affect does not predict changes in Facebook use over time and Facebook use continues to significantly predict declines in life satisfaction over time when controlling for affect. However, because participants also rated how lonely and worried they felt each time we text messaged them, we were able to test this proposal further.

We first examined whether worry or loneliness predicted changes in Facebook use over time (i.e., T_1_ worry [or T_1_ loneliness] predicting T_1–2_ Facebook use, controlling for T_0–1_ Facebook use). Worry did not predict changes in Facebook use, *B* = .04, *χ^2^* = 2.37, *p* = .12, but loneliness did, *B* = .07, *χ^2^* = 8.54, *p*<.01. The more lonely people felt at one time point, the more people used Facebook over time. Given this significant relationship, we next examined whether controlling for loneliness renders the relationship between Facebook use and changes in affective and cognitive well-being non-significant—what one would predict if Facebook use is a proxy for loneliness. This was not the case. Facebook use continued to predict declines in affective well-being, *B* = .08, *χ^2^* = 27.87, *p*<.0001, and cognitive well-being, *B* = −.012, *β* = −.126, *t*(72) = 2.34, *p* = .02, when loneliness was controlled for in each analysis. Neither worry nor loneliness interacted significantly with Facebook use to predict changes in affective or cognitive well-being (*p*s>.44).

#### Moderation

Next, we examined whether a number of theoretically relevant individual-difference variables including participants' number of Facebook Friends, their perceptions of their Facebook network support, depressive symptoms, loneliness, gender, self-esteem, time of study participation, and motivation for using Facebook (e.g., to find new friends, to share good or bad things, to obtain new information) interacted with Facebook use to predict changes in affective or cognitive well-being (Text S6). In no case did we observe any significant interactions (*p*s>.16).

#### Exploratory analyses

Although we did not have *a priori* predictions about whether Facebook use and direct social contact would interact to predict changes in affective and cognitive well-being, we nevertheless explored this issue in our final set of analyses. The results of these analyses indicated that Facebook use and direct social contact interacted significantly to predict changes in affective well-being, *B* = .002, *χ^2^* = 19.55, *p*<.0001, but not changes in cognitive well-being, *B* = .000, *β* = .129, *t*(71) = .39, *p* = .70. To understand the meaning of the former interaction, we performed simple slope analyses. These analyses indicated that the relationship between Facebook use and declines in affective well-being increased linearly with direct social contact. Specifically, whereas Facebook use did not predict significant declines in affective well-being when participants experienced low levels of direct social contact (i.e., 1 standard deviation below the sample mean for direct social contact; B = .00, χ^2^ = .04, p = .84), it did predict significant declines in well-being when participants experienced moderate levels of direct social contact (i.e., at the sample mean for direct social contact; B = .05, χ^2^ = 11.21, p<.001) and high levels of direct social contact (i.e., 1 standard deviation above the sample mean for direct social contact; B = .10, χ^2^ = 28.82, p<.0001).

## Discussion

Within a relatively short timespan, Facebook has revolutionized the way people interact. Yet, whether using Facebook predicts changes in subjective well-being over time is unknown. We addressed this issue by performing lagged analyses on experience sampled data, an approach that allowed us to take advantage of the relative timing of participants' naturally occurring behaviors and psychological states to draw inferences about their likely causal sequence [17], [18]. These analyses indicated that Facebook use predicts declines in the two components of subjective well-being: how people feel moment to moment and how satisfied they are with their lives.

Critically, we found no evidence to support two plausible alternative interpretations of these results. First, interacting with other people “directly” did not predict declines in well-being. In fact, direct social network interactions led people to feel *better* over time. This suggests that Facebook use may constitute a unique form of social network interaction that predicts impoverished well-being. Second, multiple types of evidence indicated that it was not the case that Facebook use led to declines in well-being because people are more likely to use Facebook when they feel bad—neither affect nor worry predicted Facebook use and Facebook use continued to predict significant declines in well-being when controlling for loneliness (which did predict increases in Facebook use and reductions in emotional well-being).

Would engaging in any solitary activity similarly predict declines in well-being? We suspect that they would not because people often derive pleasure from engaging in some solitary activities (e.g., exercising, reading). Supporting this view, a number of recent studies indicate that people's *perceptions* of social isolation (i.e., how lonely they feel)—a variable that we assessed in this study, which did not influence our results—are a more powerful determinant of well-being than *objective* social isolation [25]. A related question concerns whether engaging in any Internet activity (e.g., email, web surfing) would likewise predict well-being declines. Here too prior research suggests that it would not. A number of studies indicate that whether interacting with the Internet predicts changes in well-being depends on how you use it (i.e., what sites you visit) and who you interact with [26].

### Future research

Although these findings raise numerous future research questions, four stand out as most pressing. First, do these findings generalize? We concentrated on young adults in this study because they represent a core Facebook user demographic. However, examining whether these findings generalize to additional age groups is important. Future research should also examine whether these findings generalize to other online social networks. As a recent review of the Facebook literature indicated [2] “[different online social networks] have varied histories and are associated with different patterns of use, user characteristics, and social functions (p. 205).” Therefore, it is possible that the current findings may not neatly generalize to other online social networks.

Second, what mechanisms underlie the deleterious effects of Facebook usage on well-being? Some researchers have speculated that online social networking may interfere with physical activity, which has cognitive and emotional replenishing effects [27] or trigger damaging social comparisons [8], [28]. The latter idea is particularly interesting in light of the significant interaction we observed between direct social contact and Facebook use in this study—i.e., the more people interacted with other people directly, the more strongly Facebook use predicted declines in their affective well-being. If harmful social comparisons explain how Facebook use predicts declines in affective well-being, it is possible that interacting with other people directly either enhances the frequency of such comparisons or magnifies their emotional impact. Examining whether these or other mechanisms explain the relationship between Facebook usage and well-being is important both from a basic science and practical perspective.

Finally, although the analytic approach we used in this study is useful for drawing inferences about the likely causal ordering of associations between naturally occurring variables, experiments that manipulate Facebook use in daily life are needed to corroborate these findings and establish definitive causal relations. Though potentially challenging to perform—Facebook use prevalence, its centrality to young adult daily social interactions, and addictive properties may make it a difficult intervention target—such studies are important for extending this work and informing future interventions.

### Caveats

Two caveats are in order before concluding. First, although we observed statistically significant associations between Facebook usage and well-being, the sizes of these effects were relatively “small.” This should not, however, undermine their practical significance [29]. Subjective well-being is a multiply determined outcome—it is unrealistic to expect any single factor to powerfully influence it. Moreover, in addition to being consequential in its own right, subjective well-being predicts an array of mental and physical health consequences. Therefore, identifying any factor that systematically influences it is important, especially when that factor is likely to accumulate over time among large numbers of people. Facebook usage would seem to fit both of these criteria.

Second, some research suggests that asking people to indicate how good or bad they feel using a single bipolar scale, as we did in this study, can obscure interesting differences regarding whether a variable leads people to feel less positive, more negative or both less positive and more negative. Future research should administer two unipolar affect questions to assess positive and negative affect separately to address this issue.

## Concluding Comment

The human need for social connection is well established, as are the benefits that people derive from such connections [30]–[34]. On the surface, Facebook provides an invaluable resource for fulfilling such needs by allowing people to instantly connect. Rather than enhancing well-being, as frequent interactions with supportive “offline” social networks powerfully do, the current findings demonstrate that interacting with Facebook may predict the opposite result for young adults—it may undermine it.

## Supporting Information

Text S1 (DOCX)

Text S2 (DOCX)

Text S3 (DOCX)

Text S4 (DOCX)

Text S5 (DOCX)

Text S6 (DOCX)
