# Supplementary material for: Facebook Use Predicts Declines in Subjective Well-Being in Young Adults
Source: PLoS One. 2013 Aug 14;8(8):e69841. doi: 10.1371/journal.pone.0069841 (PMC3743827; doi:10.1371/journal.pone.0069841)
Supplement: Text S1 — (DOCX) [file pone.0069841.s001.docx]

Text S1: We do not imply that no longitudinal research on Facebook has been performed. Rather, no published work that we are aware of has examined how Facebook influences subjective well-being over time (i.e., how people feel and their life satisfaction).
