# Supplementary material for: Facebook Use Predicts Declines in Subjective Well-Being in Young Adults
Source: PLoS One. 2013 Aug 14;8(8):e69841. doi: 10.1371/journal.pone.0069841 (PMC3743827; doi:10.1371/journal.pone.0069841)
Supplement: Text S2 — (DOCX) [file pone.0069841.s002.docx]

Text S2: Additional measures were administered during Phase’s 1 and 2 for other purposes. The measures reported in the MS are those that were theoretically motivated.
