# Supplementary material for: Facebook Use Predicts Declines in Subjective Well-Being in Young Adults
Source: PLoS One. 2013 Aug 14;8(8):e69841. doi: 10.1371/journal.pone.0069841 (PMC3743827; doi:10.1371/journal.pone.0069841)
Supplement: Text S4 — (DOCX) [file pone.0069841.s004.docx]

Text S4: We also examined whether T_0-1_  (rather than T_1-2_) Facebook use influences T_2_ affect, controlling for T_1_ affect. Nested time-lagged analyses indicated that this was also true, *B* = .03, *χ^2^* = 4.67, *p* = .03.
