# Supplementary material for: Facebook Use Predicts Declines in Subjective Well-Being in Young Adults
Source: PLoS One. 2013 Aug 14;8(8):e69841. doi: 10.1371/journal.pone.0069841 (PMC3743827; doi:10.1371/journal.pone.0069841)
Supplement: Text S5 — (DOCX) [file pone.0069841.s005.docx]

Text S5: Some research suggests that affect fluctuates throughout the day. Replicating this work, time of day was related to affective well-being such that people reported feeling better as the day progressed (*B* = -1.06, *χ^2^* = 21.49, *p* < .0001). Controlling for time of day did not, however, substantively influence any of the results.
