# Supplementary material for: Facebook Use Predicts Declines in Subjective Well-Being in Young Adults
Source: PLoS One. 2013 Aug 14;8(8):e69841. doi: 10.1371/journal.pone.0069841 (PMC3743827; doi:10.1371/journal.pone.0069841)
Supplement: Text S6 — (DOCX) [file pone.0069841.s006.docx]

Text S6: 98% of participants reported using Facebook to “keep in touch with friends.” Therefore, we did not test for moderation with this variable.
